# Warm Steam Inhalation before Bedtime Improved Sleep Quality in Adult Men

**DOI:** 10.1155/2019/2453483

**Published:** 2019-08-25

**Authors:** Tomohisa Ichiba, Kenta Kakiuchi, Masahiro Suzuki, Makoto Uchiyama

**Affiliations:** ^1^Personal Health Care Laboratory, Kao Corporation, Tokyo, Japan; ^2^Department of Psychiatry, Nihon University School of Medicine, Tokyo, Japan

## Abstract

In humans, the inhalation of warm steam has been reported to decrease the respiratory rate. However, the effects of warm steam inhalation on sleep have not been studied closely. This study aimed to examine the effects of warm steam inhalation before bedtime on subsequent sleep quality. The participants included 17 adult men with mild sleep difficulties and anxiety. All experiments were conducted in the participants' homes. The participants were instructed to wear a warm steam-generating mask or sham mask over the nose and mouth for 15 minutes immediately before habitual bedtime and were then allowed to sleep until their habitual waking time. The functional mask provided approximately 600 mg of steam for 10 minutes and maintained an interior temperature of 38–40°C for 15 minutes. We evaluated the participants' electrocardiograms and subjective moods while wearing the mask. During sleep, electroencephalograms (EEGs) were recorded using a single-channel portable device. In the morning, each participant was instructed to report their sleep details subjectively using a visual analog scale. At bedtime, the subjects' subjective apprehension of the next day was reduced significantly under steam inhalation, compared with the sham condition. Compared to the sham condition, steam inhalation before bedtime was associated with a higher EEG delta power density during the first third of sleep episodes and better subjective sleep quality in the morning. These results suggest that safe and easy inhalation of warm steam via a steam-generating mask improves psychological relaxation and sleep.

## 1. Introduction

Pharmacotherapy for insomnia has been widely employed. However, the associated side effects and dependencies present challenges to the long-term use of hypnotic drugs [1]. Nonpharmacological treatments, such as relaxation techniques or cognitive behavior therapy for insomnia (CBT-I), are recommended as complementary and alternative therapies [2]. Various relaxation techniques are used to reduce somatic tension and treat insomnia (e.g., progressive muscle relaxation and diaphragmatic breathing) [3]. However, these relaxation techniques require training and practice to be effective and may be difficult for those with a limited range of motion. Therefore, nonpharmacological treatments are not widely used in adults with insomnia or mild sleep difficulties. Therefore, safe and easy relaxation techniques are needed to treat the large population of individuals experiencing insomnia and other sleep difficulties.

Breathing plays an important role in both physiological and psychological states and influences emotions such as anxiety, fear, sadness, and happiness [4, 5]. Moreover, therapeutic breathing techniques (e.g., biofeedback and autogenic training) have been associated with relaxation [6, 7]. We recently developed a novel breathing technique based on a disposable heat-and-steam generator (HSG) sheet to enable safe and easy inhalation of warm steam. This practice has been reported to induce psychological relaxation and decrease respiratory frequency in healthy men [8, 9] and in patients with chronic obstructive pulmonary disease (COPD) [10].

We previously reported that periocular skin warming induced psychological and physiological relaxation [11] and improved the subjective and objective quality of sleep [12, 13]. Possibly, warm steam inhalation may have similar beneficial effects on sleep quality through psychological relaxation. However, the sleep-related effects of warm steam inhalation have not been studied in detail.

In this study, we investigated whether warm steam inhalation before bedtime would improve the sleep quality in individuals with mild sleep difficulties and anxiety by promoting psychological relaxation. We monitored the electrocardiogram (ECG) results and changes in subjective status at bedtime while the participants inhaled warm steam via a mask fitted with an HSG sheet, as described in previous studies [8–10]. We then evaluated the participants' subsequent sleep quality using electroencephalograms (EEGs) and a visual analog scale (VAS).

## 2. Materials and Methods

### 2.1. Participants

We recruited, through a clinical research organization, 25 adult men with mild sleep difficulties (Pittsburgh Sleep Questionnaire Index (PSQI) [14] scores of 6–9) and anxiety (State-Trait Anxiety Inventory (STAI) [15] scores of 33–53). None of the participants had taken hypnotic medicines in the previous month, and none had previous or current physical or psychological disorders. Those who had engaged in shift work or who had a habitually short nocturnal sleep duration (<5 hours) were excluded. All participants were nonsmokers, and none habitually consumed alcohol before bedtime. Written informed consent was obtained from all study participants after they had received a detailed explanation of the experiment. Ethical approval was obtained from the Ethics Committee of Nihon University (approval number: 28–10). The study protocol was registered in the University Hospital Medical Information Network Clinical Trials Registry (UMIN-CTR registry ID: UMIN000025298) on December 16, 2016. The study itself was conducted in January and February 2017.

One participant dropped out of the because of a business trip during the study period, five participants were unable to adhere to their daily habitual sleep-wake schedule or limit alcohol consumption, and two participants misunderstood the experimental procedure. Finally, the data of 17 participants (mean ± standard deviation (SD) age: 41.2 ± 5.0 years, PSQI: 6.4 ± 0.6, Trait-STAI: 39.5 ± 6.5) were included in the analysis.

### 2.2. Experimental Design

**Figure 1:**
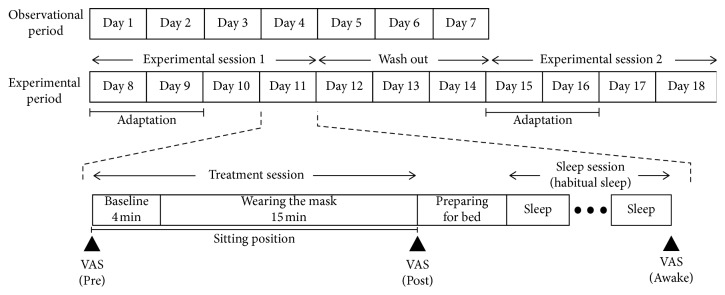
Experimental protocol. After an observational period, the experimental period comprised experimental sessions 1 and 2, separated by a 3-day interval. In each experimental session, participants performed the study according to their habitual bedtime. The first half of the experimental session was used as an adaptation period, and the second half was used for analysis.

A single-blind, placebo-controlled, randomized cross-over design was used. All experiments were performed in the participant's homes.  Figure 1 depicts the experimental protocol. First, all participants were instructed to maintain their habitual sleep-wake schedule for 7 days (observational period). After this period, participants were assigned to two experimental sessions separated by a 3-day interval. One experimental session involved the “steam-inhalation condition,” and the other involved the “sham condition.” In the steam-inhalation condition, participants used a disposable steam-generating mask (SG-mask) that covered the nose and mouth and inhaled warm steam via this mask before bedtime. In the sham condition, participants wore a non-steam sham mask (NS-mask). Each experimental session comprised 4 consecutive nights. The first 2 nights were used for adaptation, and the last 2 nights were used for analysis. The participants were required to maintain their habitual daily sleep-wake schedule throughout the experimental period and were prohibited from consuming alcohol or ingesting foods or beverages containing caffeine after dinner. They were also instructed to finish bathing or showering 1 hour before bedtime.

During each experimental session, the participants were instructed to prepare for ECG and EEG measurements before their habitual bedtime and to maintain a resting state on our provided recumbent chair for 4 minutes during ECG measurements. The participants were then asked to wear the SG- or NS-mask for 15 minutes while maintaining the same posture. During each treatment session, the participants were instructed to manage their time using an electronic timer with an alarm function. Once the 15 minutes had passed, each participant removed the mask, completed the VAS questionnaire, and laid down to sleep with a portable EEG device.

### 2.3. Mask

The SG-mask used in the present study was made of nonwoven fabric shaped in three dimensions. HSG sheets were inserted into each mask [8–10]. The mask was sealed in an aluminum package before use. Warm steam was generated through a chemical reaction of iron, water, and oxygen when the package was opened. This warm steam was applied to the skin and inhaled safely once the mask covered the nose and mouth. Our previous study showed that the mask provided approximately 600 mg of warm steam during a 10-minute period, which maintained the skin temperature underneath the mask at 38–40°C for approximately 15 minutes [8–10]. The NS-mask was composed of the same nonwoven fabric and was indistinguishable from the SG-mask. The NS-mask did not provide steam when the package was opened because the HSG sheets were inactivated. Each mask covered the nose and mouth to ensure that all breathing occurred within the mask. The masks were a prototype produced for the present study by Kao Corporation (Tokyo, Japan).

### 2.4. Evaluation of Subjective Status

In each treatment session, the subjective status was assessed before bedtime using a 100 mm VAS comprising the following seven items [16]: “How do you think the next day will be?” (an apprehension measure: 0, very pleasant; 100, very difficult), “Uneasiness” (0, very calm; 100, very uneasy), “Tension” (0, very relaxed; 100, very tense), “Nervousness” (0, not nervous at all; 100, very nervous), “Stress” (0, not stressed at all; 100, very stressed), “How do you think it will be to wake up in the morning?” (measure of difficulty in waking: 0, very easy; 100, very difficult), and “Fatigue” (0, not at all tired; 100, extremely tired). Each participant was instructed to complete the subjective status questionnaire before and after the 15-minute treatment session in accordance with how they felt at that moment. To compare subjective changes between the sham and steam-inhalation conditions, all subjective statuses were expressed relative to the subjective status obtained before the treatment.

The subjective sleep status during the sleep session was assessed using a 100 mm VAS after the final waking in the morning. The following items were used: “Sleep initiation” (0, very difficult; 100, very easy), “Sleep quality” (0, very poor sleep; 100, very good sleep), and “Feeling of being refreshed in the morning” (0, not at all; 100, extremely refreshed) [12, 13].

### 2.5. Measurement and Analysis of ECG and EEG

ECG and EEG data were recorded in the participants' homes using a single-channel portable EEG device (Brainwave Sensor ZA®; Proasist Co., Osaka, Japan) at a sampling rate of 128 Hz [17–19]. Before bedtime, participants were instructed to place the disposable Ag/AgCl surface electrodes for ECG on their chests and those for EEG in the median-frontal region in reference to the right mastoid. The raw signals were stored on an SD card and later analyzed off-line.

The R-R interval (RRI), defined as the interval between the onset times of consecutive R waves, was detected from the ECG signals, and commercial software were used to calculate a spectral analysis of the heart-rate variability (HRV) based on the RRI for each 3-minute epoch according to the maximum-entropy method (MemCalc/Win ver.2.0; GMS Co., Ltd., Tokyo, Japan). The values for the HRV bands were analyzed as follows: low frequency (LF) at 0.04–0.15 Hz and high frequency (HF) at 0.15–0.4 Hz. The ratio of LF to HF (LF/HF) was also estimated. The heart rate (HR) was calculated as 1/(RRI *∗* 60). The LF/HF ratio has been reported as a reflection primarily of the sympathetic nervous function, while the HF reflects parasympathetic nervous function. To compare changes in the HR and HRV between the sham and steam-inhalation conditions, the ratios of values from the first and second halves of the treatment session were calculated with reference to the baseline values.

According to the previously described original criteria [17–19], the sleep EEG record was divided into 30-second epochs and classified into the following sleep stages: awake, rapid-eye-movement (REM) sleep (stage N1), light non-REM (NREM) sleep (stage N2), or deep NREM sleep (stage N3). Sleep latency, sleep efficiency, and wake after sleep onset were calculated based on the sleep-stage analysis.

Spectral analyses of the EEG data were performed using a fast Fourier transform algorithm and specialized software (SleepSign-Light; KISSEI COMTEC Co., Ltd., Nagano, Japan). Power values were obtained in the following bands: delta (1.0–4.0 Hz), theta (4.0–8.0 Hz), alpha (8.0–12.0 Hz), and beta (16.0–35.0 Hz). The mean spectral power density and standard deviation (SD) were computed for the individual bands. The spectral power data for each epoch that exceeded the mean spectral power +3 SD and determined the awake stage were deemed artifacts and excluded from analysis. Next, the power values in each band were normalized to the mean power values in each band across the total sleep period. A NREM-REM cycle was defined as a NREM episode of at least 15 minutes and successive REM episode of at least 5 minutes [20]. Thus, the normalized EEG power was averaged during each NREM-REM cycle [19].

### 2.6. Statistical Analyses

General values are expressed as means ± SDs. Participant data were analyzed using nonparametric statistical tests. Statistical comparisons of conditions were performed using the Wilcoxon signed-rank test. All statistical analyses were performed using IBM SPSS Statistics 20 (IBM Corp., Armonk, NY, USA). Probability values of <0.05 were considered statistically significant.

## 3. Results

### 3.1. Subjective and Heart-Rate Changes

Table 1 presents the results of statistical comparisons and changes in seven subjective measures after treatments under the sham and steam-inhalation conditions. A significant reduction in the change in (Δ) apprehension was observed in the steam-inhalation condition relative to that in the sham condition (Table 1). Other items related to the subjective status did not differ significantly between the two conditions (Table 1). Neither the ΔHR nor the ΔHRV differed significantly between the sham and steam-inhalation conditions, although the ΔHR at first treatment and HF at the second treatment were nearly significant (Table 2).

### 3.2. EEG and Subjective Sleep Measures

Tables 3 and 4 summarize the EEG and subjective sleep measure data obtained during the sham and steam-inhalation conditions. The period of deep NREM latency was significantly shorter in the steam-inhalation condition than in the sham condition (Table 3). The subjective sleep quality reported upon waking was significantly better in the steam-inhalation condition than in the sham condition (Table 4).

### 3.3. Quantitative Sleep EEG Analyses

**Figure 2:**
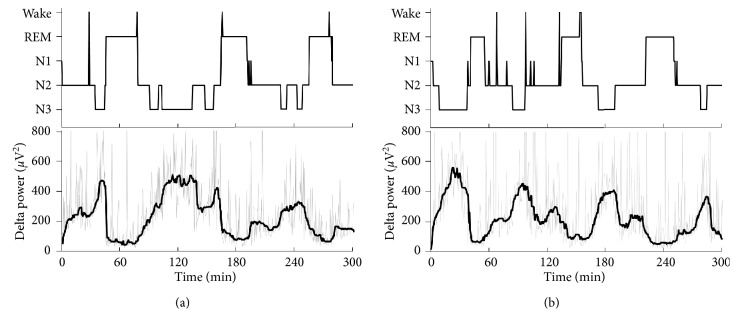
Hypnogram (upper) and delta power (lower) profiles from a representative participant. (a) Sham condition. (b) Steam-inhalation condition. W, wake; REM: rapid eye movement; N1: non-REM sleep stage N1; N2: non-REM sleep stage N2; N3: non-REM sleep stage N3.

Figure 2 presents the sleep EEG profiles of a representative participant. Notably, the deep non-REM sleep stage and delta power in the first NREM-REM cycle were more marked under the steam-inhalation condition than under the sham condition. In the first NREM-REM cycle, the delta and theta powers were enhanced significantly in the steam-inhalation condition, compared to the sham condition (Table 5). In the second and third NREM-REM cycles, none of the variables differed between the sham and steam-inhalation conditions.

## 4. Discussion

Our findings revealed that warm steam inhalation reduced the participants' apprehension before bedtime, shortened deep NREM sleep latency, and increased the sleep EEG theta and delta powers in the first third of sleep episodes. Steam inhalation also improved the subjective sleep quality upon waking when compared with the sham treatment.

Many studies have indicated that the respiratory or breathing rate is influenced by the emotional status. For example, nervousness or anxiety can cause shallow breathing and an increased respiration rate [5]. Some behavioral regimens intended to optimize breathing rate reportedly confer psychological relaxation and are widely utilized in the field of psychosomatic medicine. Previous studies have demonstrated that the use of various techniques (e.g., paced breathing [21], HRV biofeedback [22], and Zen medication [23]) to reduce the respiratory rate also enhanced the subjectively reported psychological relaxation. In the present study, psychological relaxation was achieved after using a SG-mask, although autonomic respiratory variables were not measured. Recently, properly controlled warm steam inhalation has been reported to reduce nasal resistance and alter the breathing pattern from rapid shallow breathing to slow deep breathing while achieving psychological relaxation [8–10]. These findings suggest that psychological changes might be at least partly associated with a reduction of nasal resistance and/or achievement of slow deep breathing in the present study. The sensory mechanism mediating such responses remains unclear, although the sensory branches of the trigeminal nerve might be involved.

Furthermore, we observed that the HR and HRV indicated changes associated with psychological relaxation, although the small number of participants and consequent lack of power precluded the comparison from reaching statistical significance. As warm steam inhalation may have had both physiological and psychological effects in this study, a higher mechanism associated with physiological changes may have been involved in the observed signs of psychological relaxation, such as the reduction in apprehension at bedtime.

In this study, we found that in addition to reducing subjective apprehension at bedtime, warm steam inhalation shortened the deep NREM latency and increased the EEG delta power in the first third of sleep episodes compared with the sham condition. As the participants were instructed to remove the NS- or SG-mask immediately after the 15-minute treatment, fill out the questions on subjective states, and then retire, the reduction in subjective apprehension associated with the steam-inhalation condition might have influenced the subsequent sleep status. Anxiety at bedtime has been associated negatively with the amount of slow wave sleep [16]. Furthermore, we have recently reported that warming of the periocular skin region to approximately 40°C before bedtime was associated with an increase in relaxed feelings at bedtime and an increase in EEG delta power value in the early hours of subsequent sleep [13]. Together with those previous findings, our present results support the notion that psychological relaxation after warm steam inhalation might contribute to a propensity to sleep and the amount of slow wave sleep in subjects with mild sleep difficulties.

In the present study, the subjective sleep quality at the time of waking was also improved by warm steam inhalation before bedtime. However, the direct effects of this treatment on sleep were limited to the early hours of sleep. Huber et al. have reported that slow wave activity in early sleep was associated with the recovery of brain function [24]. Recently, we found that psychological and physiological relaxation via local skin warming in the periocular area and back of the neck increased delta power early in the sleep episode and improved the subjective sleep quality upon waking [13]. The present finding that an increase in delta sleep in the early sleep episode after bedtime steam inhalation improved the subjective sleep quality upon waking may be comparable to the findings of previous studies [13]. Thus, enhanced deep sleep in the early sleep episode may play a key role in improving the subjective sleep quality at waking, as suggested in previous basic studies [24].

This study had several limitations in the present study. First, the HR and HRV did not reach statistical significance. These results may be attributable to a lack of power caused by the small number of participants. Further studies with a large number of participants are required to clarify the relationship between physiological relaxation and steam inhalation. Second, the experimental studies were conducted in the participants' homes as described in a previous study [13]. Although participants in this study were instructed to maintain their habitual sleep-wake schedule and sleep environment, potential confounding factors such as the room temperature and/or humidity, illumination, and noise levels might have influenced physiological changes such as the HR and HRV and sleep. Further investigation is needed to clarify the effects of steam inhalation on the physiological state and sleep quality in an environment minimized these potential confounding effects. Third, although participants could not distinguish the SG- and NS-masks by appearance alone, they could feel the difference between the masks during use. Accordingly, the results may have been confounded by the sensations experienced when wearing the mask. Fourth, the effect of the respiratory rate on steam inhalation was not investigated in the present study. Future studies are needed to clarify the relationship between changes in respiratory rates and sleep.

## 5. Conclusion

According to the study findings, warm steam inhalation before bedtime induced psychological relaxation and increased deep sleep in the early sleep episode, leading to an improved subjective sleep quality in participants with mild sleep difficulties and anxiety. These results suggest that safe and easy inhalation of warm steam via a steam-generating mask may have favorable effects on relaxation and sleep.

## Figures and Tables

**Table 1:** Subjective changes during sham and steam-inhalation conditions.

	Sham	Steam inhalation	*p*
Δ apprehension	0.0 ± 7.2	–5.7 ± 7.3	0.026
Δ uneasiness	–1.4 ± 8.2	–2.3 ± 6.9	0.507
Δ tension	–2.4 ± 11.9	–3.7 ± 8.7	0.981
Δ nervousness	–3.5 ± 13.1	–6.3 ± 12.0	0.407
Δ stress	–3.7 ± 11.3	–3.5 ± 12.0	0.408
Δ difficulty waking up	–2.3 ± 11.3	–3.1 ± 6.7	0.756
Δ fatigue	–5.2 ± 8.8	–5.5 ± 9.6	0.740

Values are expressed as means ± standard deviations. Comparisons are relative to the sham condition (Wilcoxon signed-rank test).

**Table 2:** HR and HRV during sham and steam-inhalation conditions.

	First treatment			Second treatment		
	Sham	Steam inhalation	*p*	Sham	Steam inhalation	*p*
HR (bpm)	–2.4 ± 3.8	–4.0 ± 3.2	0.075	–3.7 ± 3.8	–4.0 ± 5.6	0.600
HF (%)	129 ± 48	162 ± 76	0.263	125 ± 47	184 ± 99	0.087
LF/HF (%)	112 ± 46	132 ± 110	0.972	131 ± 97	193 ± 152	0.552

Values are expressed as means ± standard deviations. Comparisons are relative to the sham condition (Wilcoxon signed-rank test). HR: heart rate; HF: high frequency; LF: low frequency; LF/HF: ratio of LF to HF.

**Table 3:** Comparison of sleep parameters in the two conditions.

	Sham	Steam inhalation	*p*
Bedtime (h : min)	0 : 46 ± 0 : 51	0 : 45 ± 0 : 54	0.807
Wake time (h : min)	7 : 02 ± 0 : 38	6 : 59 ± 0 : 26	0.221
SPT (min)	380.1 ± 60.7	370.5 ± 63.5	0.221
TST (min)	348.0 ± 71.3	342.8 ± 66.9	0.311
Sleep latency (min)	20.2 ± 25.9	14.1 ± 22.5	0.600
WASO (min)	11.9 ± 10.7	13.6 ± 23.8	0.861
Sleep efficiency (%)	91.2 ± 8.5	92.4 ± 7.8	0.807
Deep NREM (%)	20.2 ± 6.4	22.2 ± 10.1	0.753
Deep NREM latency (min)	23.8 ± 16.9	17.7 ± 12.1	0.045
REM latency (min)	48.6 ± 22.8	67.2 ± 34.2	0.196

Values are expressed as means ± standard deviations. Comparisons are relative to the sham condition (Wilcoxon signed-rank test). SPT: sleep period time; TST: total sleep time; WASO: wake after sleep onset; REM: rapid eye movement; NREM: non-REM.

**Table 4:** Subjective sleep scores in the morning.

	Sham	Steam inhalation	*p*
Sleep initiation (mm) (0: poor, 100: good)	58.6 ± 21.1	66.0 ± 16.9	0.058
Sleep quality (mm) (0: low, 100: good)	49.4 ± 13.3	59.5 ± 16.0	0.005
Feeling of being refreshed (mm) (0: poor, 100: good)	50.2 ± 12.1	52.7 ± 21.3	0.569

Values are expressed as means ± standard deviations. Comparisons are relative to the control session (Wilcoxon signed-rank test).

**Table 5:** Courses of sleep EEG power in the delta, theta, alpha, and beta bands in the sham and steam-inhalation conditions.

Normalized EEG	First NREM-REM cycle			Second NREM-REM cycle			Third NREM-REM cycle		
	Sham	Steam	*p*	Sham	Steam	*p*	Sham	Steam	*p*
Delta (%) (1–4 Hz)	89.6 ± 36.0	121.0 ± 45.1	0.039	105.7 ± 34.5	103.5 ± 31.3	0.972	101.3 ± 45.9	93.2 ± 22.6	0.552
Theta (%) (4–8 Hz)	90.9 ± 40.1	125.1 ± 58.0	0.028	145.0 ± 44.2	135.9 ± 87.2	0.421	150.2 ± 71.6	187.8 ± 95.7	0.422
Alpha (%) (8–12 Hz)	73.8 ± 32.6	98.2 ± 44.4	0.064	104.8 ± 33.9	104.5 ± 36.0	0.917	95.2 ± 42.0	94.2 ± 23.1	0.807
Beta (%) (16–35 Hz)	70.7 ± 36.1	94.7 ± 61.2	0.075	95.7 ± 34.9	98.6 ± 39.5	0.861	106.3 ± 46.8	101.2 ± 32.3	0.753

Values are expressed as means ± standard deviations. Comparisons are relative to the sham condition (Wilcoxon signed-rank test).

## Data Availability

All data used to support the findings of this study are available from the corresponding author upon request.
